# Identifying best modelling practices for tobacco control policy simulations: a systematic review and a novel quality assessment framework

**DOI:** 10.1136/tobaccocontrol-2021-056825

**Published:** 2022-01-11

**Authors:** Vincy Huang, Anna Head, Lirije Hyseni, Martin O'Flaherty, Iain Buchan, Simon Capewell, Chris Kypridemos

**Affiliations:** Department of Public Health, Policy and Systems, University of Liverpool, Liverpool, UK

**Keywords:** public policy, smoking caused disease, socioeconomic status

## Abstract

**Background:**

Policy simulation models (PSMs) have been used extensively to shape health policies before real-world implementation and evaluate post-implementation impact. This systematic review aimed to examine best practices, identify common pitfalls in tobacco control PSMs and propose a modelling quality assessment framework.

**Methods:**

We searched five databases to identify eligible publications from July 2013 to August 2019. We additionally included papers from Feirman *et al* for studies before July 2013. Tobacco control PSMs that project tobacco use and tobacco-related outcomes from smoking policies were included. We extracted model inputs, structure and outputs data for models used in two or more included papers. Using our proposed quality assessment framework, we scored these models on population representativeness, policy effectiveness evidence, simulated smoking histories, included smoking-related diseases, exposure-outcome lag time, transparency, sensitivity analysis, validation and equity.

**Findings:**

We found 146 eligible papers and 25 distinct models. Most models used population data from public or administrative registries, and all performed sensitivity analysis. However, smoking behaviour was commonly modelled into crude categories of smoking status. Eight models only presented overall changes in mortality rather than explicitly considering smoking-related diseases. Only four models reported impacts on health inequalities, and none offered the source code. Overall, the higher scored models achieved higher citation rates.

**Conclusions:**

While fragments of good practices were widespread across the reviewed PSMs, only a few included a ‘critical mass’ of the good practices specified in our quality assessment framework. This framework might, therefore, potentially serve as a benchmark and support sharing of good modelling practices.

## Introduction

Since 2020, it became evident that COVID-19 modelling had influenced, and on occasions dictated, disease control policies to shape the subsequent course of the pandemic.^1^ For decades before this publicity, policy simulation models (PSMs) have been applied to inform evidence-based health policymaking and had contributed to many successful tobacco control policies.^2^

Various actions have been taken to end the tobacco pandemic, which killed over 100 million people worldwide during the 20th century.^3^ These actions notably include policies targeting the accessibility, acceptability and affordability of tobacco products. Tobacco control models have been used extensively to shape such policies, both prior to real-world implementation and also to evaluate post-implementation impact.^4^

Two recent systematic reviews identified a plethora of tobacco control models intended for policymaking and policy evaluation.^5–7^ This is a very active research area, reflecting an explosion of available data, novel methodologies and low-cost, widely available computational power.^8^ However, this plethora of independently developed models may represent an unnecessary effort in replication compared with a more collaborative approach. Second, neither previous systematic review examined model quality, which perhaps reflects a lack of an appropriate quality assessment framework for simulation models.

Although several publicly available quality assessment tools exist, including Consolidated Health Economic Evaluation Reporting Standards (CHEERS) checklist,^9^ Grading of Recommendations, Assessment, Development and Evaluations (GRADE),^10^ and the National Institute for Health and Care Excellence (NICE) Methodology Guide quality checklist,^11^ none appear well suited for the diversity of tobacco control models. The NICE and CHEERS checklists are designed mainly to evaluate economics models, while the GRADE guideline focuses mainly on evidence certainty and is not topic specific.

The lack of such an applicable framework partly reflects the fast evolution of modelling approaches, the multidisciplinary nature of modelling and the multitude of questions models are asked to address. While developing a generic quality assessment framework for simulation models appears challenging, developing a domain-specific one for tobacco control simulation models might represent a more feasible first step.

We, therefore, defined two aims for this study:

1. To assess the modelling practices used in tobacco control PSMs (reviewing both best practices and common limitations).
2. To produce a quality assessment framework appropriate for tobacco control PSMs to potentially improve future policy modelling practices.

## Methods

### Study design

We systematically reviewed the published tobacco control PSMs, particularly evaluating their methodological strengths and weaknesses. We then critically appraised and compared their modelling practices with an ideal but feasible tobacco control PSM prototype.

We report the results following the Synthesis Without Meta-Analysis statement (online supplemental table S1),^12^ and present our findings in compliance with the Preferred Reporting Items for Systematic Reviews and Meta-Analysis statement.^13^

10.1136/tobaccocontrol-2021-056825.supp1 Supplementary data

### Definitions

**PSMs:** quantitative frameworks that integrate evidence from cross-disciplinary sources to estimate the impact of existing or planned policies.

**Tobacco control PSMs:** PSMs that estimate existing or planned tobacco control policies impact.

**Smoking-related diseases:** diseases widely accepted to be causally linked to smoking, including chronic obstructive pulmonary disease (COPD), cardiovascular disease and common cancers.

**External validation:** comparing the model result with actual observed data not used as model inputs.^14 15^

**Cross-validation:** comparison of results between models which address the same problem.^14 15^

**Sensitivity analysis:** studying the model output changes caused by varying model inputs.^14 15^

### Search strategy

We included the studies in the Feirman *et al* systematic review (to July 2013)^6^ and extended the search strategy to cover the period to September 2019.

We searched five electronic databases (Embase, EconLit, PsycINFO, PubMed and CINAHL Plus). The search keywords for five databases are detailed in online supplemental text S1. We also scanned the reference lists of all included studies for potential additional papers.

### Study selection and inclusion criteria

Inclusion criteria:

1. Referred to any tobacco product or tobacco use.
2. Contained peer-reviewed tobacco control PSMs that projected tobacco use and tobacco-related outcomes from tobacco control policy scenarios.
3. The model was reported in at least two peer-reviewed studies.
4. Full text in English.

We assessed the retrieved studies using the Participants, Interventions, Comparators, Outcomes and Study design approach (online supplemental table S2).

Two reviewers (VH and AH) independently screened titles and abstracts for eligibility using the inclusion and exclusion criteria, then screened the full text of all potential eligible papers. A third reviewer (CK) with modelling expertise was consulted to resolve any discrepancies. We used Zotero, reference management software, for screening.

We registered the protocol for our study with PROSPERO (CRD42020178146) and published it separately.^16^

### Data extraction

We used a predefined and piloted data extraction form (online supplemental table S3) to extract study information on:

1. General information (ie, model name, code license, conflict of interest (COI)).
2. Model simulation methods.
3. Modelled population sociodemographic characteristics (ie, age, gender, ethnicity/race, socioeconomic status).
4. Risk factors.
5. Included diseases.
6. Data sources used.
7. Model outcome types (ie, health, economics).
8. Model checking, transparency, validation and calibration.
9. Model limitations reported.

We assessed data extraction quality by allowing a second reviewer to double-check 50% of the extraction forms for accuracy and completeness.

### Evidence synthesis

We grouped the extracted study data by model name when reported or by the first author of the earliest publication.

We critically reviewed model data inputs, epidemiological principles, assumptions, transparency and whether they reported (a) relevant sources of parametric uncertainty, (b) potential limitations, (c) model validations and sensitivity analyses, and (d) technical documentation.

### A proof-of-concept quality assessment framework

We then developed a simple quality assessment framework for model inputs, structure and outputs based on potential Good Modelling Practices (detailed in online supplemental text S2).

One point was given when each of the described criteria below was met:

- **Population:** model population data are representative of the population that the modelled policies will apply to.
- **Policy effectiveness**: the policy effectiveness data were extracted from empirical evidence.
- **Smoking status**: the model captured the cumulative effect of smoking (smoking intensity, smoking history, quitting age, etc).
- **Smoking-related diseases**: the model estimated the effect on the majority of important smoking-related diseases (quantifying both morbidity and mortality).
- **Lag time**: the model explicitly captured the time lag between exposure and disease onset.
- **Transparency:** technical or non-technical documents available to provide model transparency.
- **Uncertainty/sensitivity analysis** performed and reported.
- **Validation**: the model was validated.
- **Equity:** the model explored the equity impact of policies.

## Results

**Figure 1:**
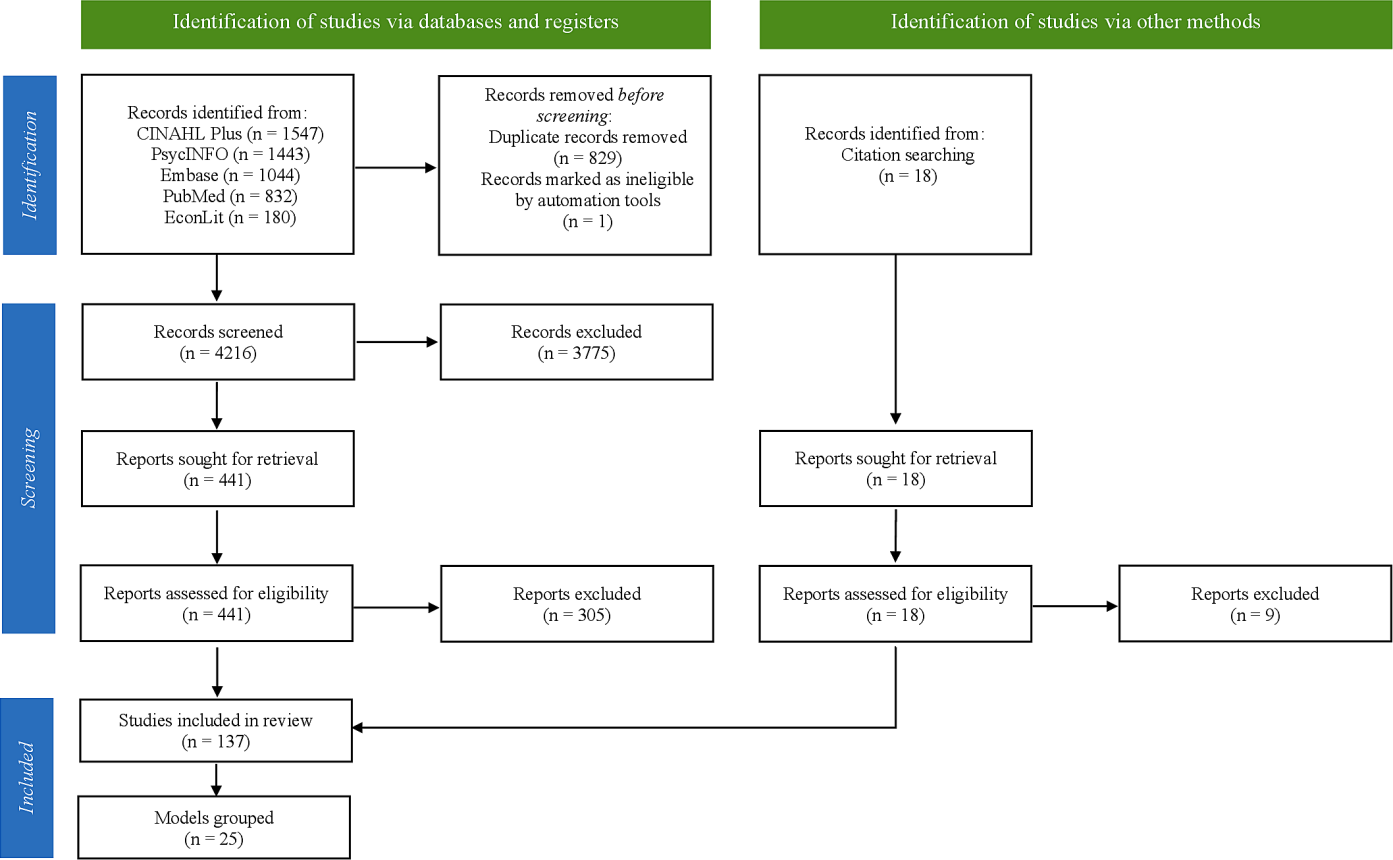
An adapted Preferred Reporting Items for Systematic Reviews and Meta-Analysis flow chart of identified studies.

The search initially identified 5046 articles. After removing duplicates and screening titles and abstracts, 441 articles were eligible for full-text review. In total, 146 studies met the inclusion criteria and were included for data extraction, including 9 additional studies identified from included studies’ reference lists (figure 1).^17–25^

We identified a total of 25 eligible tobacco control PSMs (table 1 and online supplemental text S3). Five models were used by only one paper in our searches. Nevertheless, we included them in our study because they were also used in papers published before July 2013, as identified in the Feirman *et al* review. The five models were Chronic Disease Model (CDM), Coronary Heart Disease Policy (CHD Policy) Model, Lung Cancer Policy Model (LCPM), Mendez model and Mejia model.

**Table 1:** Model summary (in descending order of the number of peer-reviewed articles)

**Model name/first author: SimSmoke** **Model type (self-reported):** discrete Markov model, macrosimulation **Risk factors included (smoking status provided with details):** smoking status (never, former, current), years since quitting **Diseases:** NA **Outcomes:** mortality, smoking prevalence, maternal and child health outcomes (smoking-attributable low birth weight, preterm births and sudden infant death syndrome) cases, uncertainty **Sensitivity analysis:** performed sensitivity analysis **Validation:** External validation **Number of peer-reviewed articles in this search:** 18 **Related papers:** 24 26–42	**Model name/first author: Burden of Disease Epidemiology, Equity and Economics model** **Model type (self-reported):** a proportional multistate life-table, macrosimulation, Markov model **Risk factors included (smoking status provided with details):** smoking status (never, former, current) **Diseases:** 16 diseases—CHD, stroke, COPD, lower respiratory tract infection, and multiple cancers: lung, oesophageal, stomach, liver, head and neck, pancreas, cervical, bladder, kidney, endometrial, melanoma, and thyroid (with smoking protecting against the latter three cancers) **Outcomes:** equity, health-systems cost-savings, smoking prevalence, QALYs gained, health-adjusted life years, uncertainty **Sensitivity analysis:** PSA **Validation:** cross-validation, external validation **Number of peer-reviewed articles in this search:** 11 **Related papers:** 20 25 43–51
**Model name/first author: IMPACT** **Model type (self-reported):** cell-based model **Risk factors included (smoking status provided with details):** blood pressure, cholesterol, diabetes, fruit and vegetable, smoking status (never smoker, long-term ex-smoker, recent ex-smoker, current smoker), salt intake, saturated fat intake, BMI, physical activities **Diseases:** CHD, T2DM **Outcomes:** equity, CHD mortality, smoking prevalence, life-years gained, uncertainty **Sensitivity analysis:** Monte Carlo simulation **Validation:** external validation **Number of peer-reviewed articles in this search:** 6 **Related papers:** 23 107–111	**Model name/first author: extended cost-effectiveness analysis (ECEA) tobacco tax model** **Model type (self-reported):** ECEA **Risk factors included (smoking status provided with details):** smoking prevalence, number of cigarettes smoked daily; age at quitting **Diseases:** COPD, heart disease, stroke, lung cancer, bladder cancer **Outcomes:** disease treatment costs, averted premature death, life-years gained, additional revenues generated, equity, uncertainty **Sensitivity analysis:** one-way, Monte Carlo simulation **Validation:** validated model **Number of peer-reviewed articles in this search:** 5 **Related papers:** 70–74
**Model name/first author: EQUIPTMOD** **Model type (self-reported):** Markov state-transition cohort model, macrosimulation **Risk factors included (smoking status provided with details):** smoking status (former, current) **Diseases:** COPD, CHD, stroke, lung cancer **Outcomes:** cost, ROI, ICER, QALY **Sensitivity analysis:** univariate, others **Validation:** no model validation **Number of peer-reviewed articles in this search:** 5 **Related papers:** 112–116	**Model name/first author: DYNAMO-HIA model** **Model type (self-reported):** macrosimulation, Markov-based life-table **Risk factors included (smoking status provided with details):** alcohol intake, BMI, smoking status, secondhand smoking **Diseases:** COPD, IHD, stroke, cancers, T2DM **Outcomes:** mortality, morbidity, morbidity-free years, life expectancy, number of deaths **Sensitivity analysis:** performed sensitivity analysis **Validation:** no model validation **Number of peer-reviewed articles in this search:** 5 **Related papers:** 94–98
**Model name/first author: Benefits of Smoking Cessation on Outcomes model** **Model type (self-reported):** discrete-time Markov model **Risk factors included (smoking status provided with details):** smoker, recent quitter and long-term quitter **Diseases:** COPD, CHD, stroke, lung cancer, asthma exacerbation, chronic obstructive lung diseases **Outcomes:** total morbidity and mortality, economics impact **Sensitivity analysis:** one-way, PSA **Validation:**no model validation **Number of peer-reviewed articles in this search:** 4 **Related papers:** 63–66	**Model name/first author: Jiménez model** **Model type (self-reported):** closed cohort Markov model **Risk factors included (smoking status provided with details):** smoking status, willingness to quit history **Diseases:** COPD, CVD, T2DM **Outcomes:** incremental cost-savings, number of quitters **Sensitivity analysis:** univariate sensitivity analysis **Validation:** internal validation **Number of peer-reviewed articles in this search:** 3 **Related papers:** 89–91
**Model name/first author: Johansson model** **Model type (self-reported):** a Markov model **Risk factors included (smoking status provided with details):** smoking status (former, current) **Diseases:** COPD, CHD, stroke, cancers **Outcomes:** QALY, life years lost, cost, uncertainty **Sensitivity analysis:** univariable, multivariable, PSA **Validation:** external validation **Number of peer-reviewed articles in this search:** 3 **Related papers:** 17 57 58	**Model name/first author: Prevention Impacts Simulation Model** **Model type (self-reported):** system dynamics model **Risk factors included (smoking status provided with details):** blood pressure, cholesterol, secondhand smoking, obesity, psychological distress, fruit and vegetable, smoking status (never smoker, long-term ex-smoker, recent ex-smoker, current smoker), blood glucose categories (normal, pre-diabetic, diabetic), periodontal disease, sleep apnoea, small particulate air pollution and inadequate use of aspirin for primary prevention **Diseases:** CVD **Outcomes:** mortality and morbidity, healthcare cost, productivity loss, uncertainty **Sensitivity analysis:** PSA **Validation:** external validation **Number of peer-reviewed articles in this search:** 3 **Related papers:** 75–77
**Model name/first author: Baker model** **Model type (self-reported):** closed cohort Markov model **Risk factors included (smoking status provided with details):** eligible smoker, ineligible smoker, initial quitter, successful quitter **Diseases:** NA **Outcomes:** number of quitters, morbidity, mortality, medical expenditures **Sensitivity analysis:** univariate, multivariable analyses **Validation:** no model validation **Number of peer-reviewed articles in this search:** 2 **Related papers:** 99 100	**Model name/first author: Barnett model** **Model type (self-reported):** a Markov model **Risk factors included (smoking status provided with details):** smoking status (former, current) **Diseases:** NA **Outcomes:** mortality, healthcare cost, QALY, uncertainty **Sensitivity analysis:** one-way, PSA **Validation:** no model validation **Number of peer-reviewed articles in this search:** 2 **Related papers:** 59 60
**Model name/first author: Cantor model** **Model type (self-reported):** decision-analytical model **Risk factors included (smoking status provided with details):** smoking status **Diseases:** NA **Outcomes:** cost, QALY **Sensitivity analysis:** one-way, two-way uncertainty analyses **Validation:** no model validation **Number of peer-reviewed articles in this search:** 2 **Related papers:** 61 62	**Model name/first author: Chevreul model** **Model type (self-reported):** Markov state transition model **Risk factors included (smoking status provided with details):** smoking status (smoker, former smoker), diagnosed with either lung cancer, COPD or CVD such as stroke or coronary artery disease and dead (smoker: ≥1 cigarette/day) **Diseases:** COPD, CVD, lung cancer **Outcomes:** ICER **Sensitivity analysis:** deterministic sensitivity analysis, Monte Carlo simulation **Validation:** internal validation, external validation **Number of peer-reviewed articles in this search:** 2 **Related papers:** 101 102
**Model name/first author: Cost-Effectiveness of Preventing AIDS Complications-US model** **Model type (self-reported):** microsimulation **Risk factors included (smoking status provided with details):** smoking intensity (packs/day)—heavy/moderate/light, CD4+T cell count, viral load, history of opportunistic disease and antiretroviral treatment use **Diseases:** lung cancer **Outcomes:** life expectancy, mortality **Sensitivity analysis:** two-way **Validation:** internal validation, external validation and cross-validation **Number of peer-reviewed articles in this search:** 2 **Related papers:** 92 93	**Model name/first author: ModelHealth: Tobacco** **Model type (self-reported):** microsimulation **Risk factors included (smoking status provided with details):** smoking status (never, former, current) **Diseases:** CVD, stroke, lung cancer, respiratory disease **Outcomes:** medical cost, hospitalisation, mortality and morbidity, productivity loss, QALY, smoking prevalence **Sensitivity analysis:** one-way **Validation:** internal validation, external validation **Number of peer-reviewed articles in this search:** 2 **Related papers:** 103 104
**Model name/first author: Parrott model** **Model type (self-reported):** decision tree **Risk factors included (smoking status provided with details):** childhood exposure to maternal smoking, smoking status (current, former) **Diseases:** COPD, CHD, stroke, lung cancer, asthma, pregnancy-related (placental abruption, ectopic pregnancy, pre-eclampsia, placenta previa and miscarriage, infant morbidities: low infant birth weight, stillbirth, premature birth) **Outcomes:** ICER, QALY, uncertainty **Sensitivity analysis:** PSA **Validation:** no model validation **Number of peer-reviewed articles in this search:** 2 **Related papers:** 67 68	**Model name/first author: Population Health Impact Model** **Risk factors included (smoking status provided with details):** never tobacco users, former tobacco users, current cigarette smokers, current cMRTP users, current dual users **Diseases:** COPD, IHD, stroke, lung cancer **Outcomes:** mortality, cMRTP uptake **Sensitivity analysis:** performed sensitivity analysis **Validation:** model validated **Number of peer-reviewed articles in this search:** 2 **Related papers:** 18 52
**Model name/first author: Tobacco Town** **Model type (self-reported):** agent-based model **Risk factors included (smoking status provided with details):** smoking intensity (cigarettes/day) **Diseases:** NA **Outcomes:** cost, tobacco purchase behaviour **Sensitivity analysis:** performed sensitivity analysis **Validation:** no model validation **Number of peer-reviewed articles in this search:** 2 **Related papers:** 79 80	**Model name/first author: UK Health Forum simulation** **Model type (self-reported):** microsimulation **Risk factors included (smoking status provided with details):** smoking status (never, former, current) **Diseases:** COPD, CHD, stroke, 14 cancers **Outcomes:** cost, morbidity, smoking prevalence, uncertainty **Sensitivity analysis:** performed sensitivity analysis **Validation:** no model validation **Number of peer-reviewed articles in this search:** 2 **Related papers:** 81 82
**Model name/first author: Chronic Disease Model** **Model type (self-reported):** closed cohort multistate Markov model **Risk factors included (smoking status provided with details):** smoking status (never, former, current) **Diseases:** acute myocardial infarction, chronic heart failure, COPD, stroke (CVA), T2DM, and cancer of the lung, stomach, oesophagus, larynx, bladder, kidney, pancreas, and oral cavity **Outcomes:** cost, QALY, number of quitters **Sensitivity analysis:** one-way **Validation:** no model validation **Number of peer-reviewed articles in this search:** 1 **Related papers:** 117	**Model name/first author: Chronic Heart Disease Policy Model** **Model type (self-reported):** state-transition (Markov) computer-simulation model **Risk factors included (smoking status provided with details):** active smoker or secondhand smoke exposure, systolic blood pressure, BMI, level of high-density lipoprotein cholesterol, level of low-density lipoprotein, diabetes **Diseases:** CHD and stroke **Outcomes:** CHD incidence, prevalence, mortality, costs, uncertainty **Sensitivity analysis:** Monte Carlo simulations **Validation:** no model validation **Number of peer-reviewed articles in this search:** 1 **Related papers:** 118
**Model name/first author: Lung Cancer Policy Model** **Model type (self-reported):** state-transition microsimulation model **Risk factors included (smoking status provided with details):** smoking history (length of time a person smoked and cigarettes smoked per day) **Diseases:** three cancers from any of the following five lung cancer cell types: adenocarcinoma (including adenocarcinoma in situ), large cell, squamous cell, small cell and other **Outcomes:** mortality rate and cost-effectiveness **Sensitivity analysis:** performed sensitivity analysis **Validation:** model validated **Number of peer-reviewed articles in this search:** 1 **Related papers:** 69	**Model name/first author: Mendez model** **Model type (self-reported):** Excel-based Markov model **Risk factors included (smoking status provided with details):** smoking status (never, former, current) **Diseases:** NA **Outcomes:** cost, DALY, smoking prevalence **Sensitivity analysis:** PSA **Validation:** model validated **Number of peer-reviewed articles in this search:** 1 **Related papers:** 78*
**Model name/first author: Mejia model** **Model type (self-reported):** decision tree model used in Monte Carlo simulations **Risk factors included (smoking status provided with details):** smoking status (current cigarette user, current e-cigarette, dual user) **Diseases:** NA **Outcomes:** morbidity, uncertainty **Sensitivity analysis:** performed sensitivity analysis **Validation:** no model validation **Number of peer-reviewed articles in this search:** 1 **Related papers:** 83	

*Paper mentioned that this simulation model is based on the model developed by Mendez, Warner and Courant.

BMI, body mass index; CHD, coronary heart disease; cMRTP, candidate modified risk tobacco products; COPD, chronic obstructive pulmonary disease; CVA, cerebrovascular accident; CVD, cardiovascular disease; DALY, disability-adjusted life year; ICER, incremental cost-effectiveness ratio; IHD, ischaemic heart disease; NA, not available; PSA, probabilistic sensitivity analysis; QALY, quality-adjusted life year; ROI, return on investment; T2DM, type 2 diabetes mellitus.

The SimSmoke model appeared to be the most used model with 18 peer-reviewed studies,^24 26–42^ and the Burden of Disease Epidemiology, Equity and Economics (BODE^3^) model ranked second with 11 peer-reviewed studies.^20 25 43–51^

Cohort (macro)simulation and microsimulation approaches were the most used self-reported methodologies. Agent-based modelling was used in just one model (Tobacco Town), likewise system dynamics in Prevention Impacts Simulation Model.

**Figure 2:**
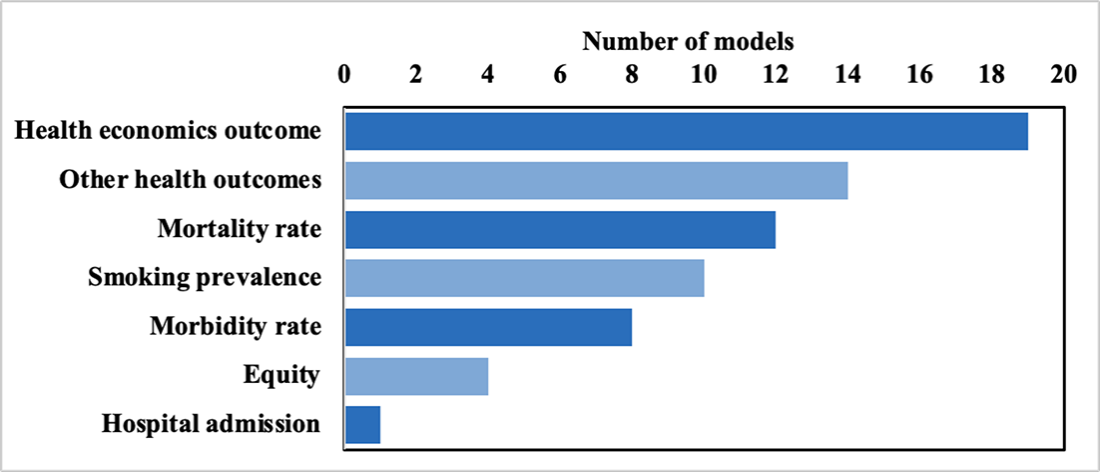
Occurrence of model outcome types (some models included more than one output type).

The diversity of model outcomes reflected the wide range of model purposes. Nineteen models reported health economics outcomes, 22 reported health measures including mortality or morbidity, with just one (ModelHealth: Tobacco) reporting hospital admissions (figure 2, online supplemental table S4). Only four models reported the policy impact on equity: BODE^3^, CDM, extended cost-effectiveness analysis (ECEA) tobacco tax model and IMPACT.

Of the eligible 146 studies, 5 tobacco industry-funded studies reported COI or commercial funding.^52–56^ However, we did not further investigate inaccurate or incomplete COI reporting.

### Good Modelling Practices

#### Model inputs

Twenty-one of the 25 models appropriately used population data from public or administrative registries. Conversely, three used information from randomised controlled trial (RCT) participants that are rarely population representative.^17 57–62^

Eleven out of 25 models used systematic reviews or meta-analyses to inform policy effectiveness in the model. Six models used treatment-specific RCT values to estimate policy effectiveness.^17 57–69^ Five models assumed the policy effectiveness by project teams or expert opinions.^18 24 26–42 52 70–78^ In particular, SimSmoke, the most frequently referenced model, used the policy effect size provided by experts, likewise the ECEA tobacco tax model. Furthermore, three models calculated policy effectiveness from government reports or surveys.^79–83^ Similarly, Population Health Impact Model estimated policy effectiveness by simple assumptions.

#### Model structure

Abiding by fundamental epidemiological principles, a tobacco simulation model structure should ideally aim to: (a) capture the cumulative effect of smoking (ie, for lung cancer and COPD^84–86^), including the intensity, duration, initiation and cessation; (b) estimate the effect on the main smoking-related diseases (ideally including both morbidity and mortality)^87^; (c) capture the time lag between exposure and disease risk^87^; (d) be transparent.^14 88^

Six models simulated smoking histories (including pack-years, pack-days) or quitting histories.^24 26–42 69–74 79 80 89–93^ A further 19 models considered smoking only as a categorical exposure (ie, never/ex/current smoker).

Lag time was reported in 11 out of 25.^17 18 20 24–52 57 58 75–77 81 82 92–104^ These models either estimated relative risk decline by time since cessation or cost decay by quit years. The remaining models did not report any considerations on the effect of time since cessation.

**Figure 3:**
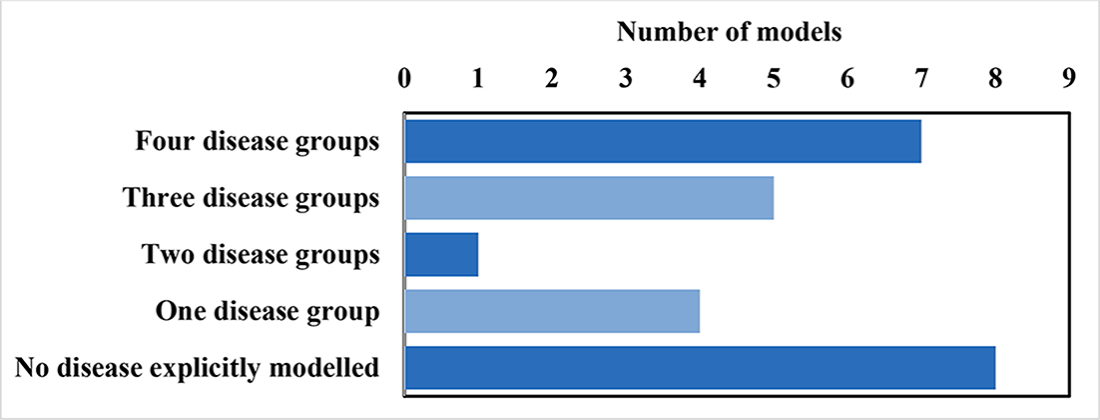
Occurrence of number of disease groups simulated by models. Considered disease groups: cancers, chronic obstructive pulmonary disease, cardiovascular disease and any other smoking-related diseases. The models with no diseases explicitly modelled either calculated all-cause mortality directly from smoking status or used the number of smokers or non-smokers without explicitly modelling disease pathways.

We summarised the number of diseases included in each model in figure 3 and online supplemental table S5. Models varied in how well they reflected epidemiology pathways (online supplemental table S6). Seventeen used smoking-related diseases to generate smoking-related outcomes. Two models calculated all-cause mortality directly from smoking status. The remaining six models estimated their outcomes directly, using the number of smokers or non-smokers, without explicitly modelling disease pathways.

#### Transparency

Nineteen models provided model documentation to explain model technical details for readers (all except Benefits of Smoking Cessation on Outcomes model, Baker model, Cantor model, Chevreul model, CHD Policy Model and Jiménez model). Some models provided detailed model information. One of the SimSmoke models, in particular, provided a detailed data source and modelling diagram.^105^ However, none of the models provided the source code or the pseudo-code of their algorithms.

#### Model output

Finally, existing modelling guidelines recommend model validation, propagation of uncertainty and sensitivity analysis.^88 106^

First, 11 of 25 models reported result uncertainty. Furthermore, the SimSmoke model reported uncertainty in some studies but not in others. The types of uncertainty that were reflected in the reported uncertainty intervals varied widely.

**Figure 4:**
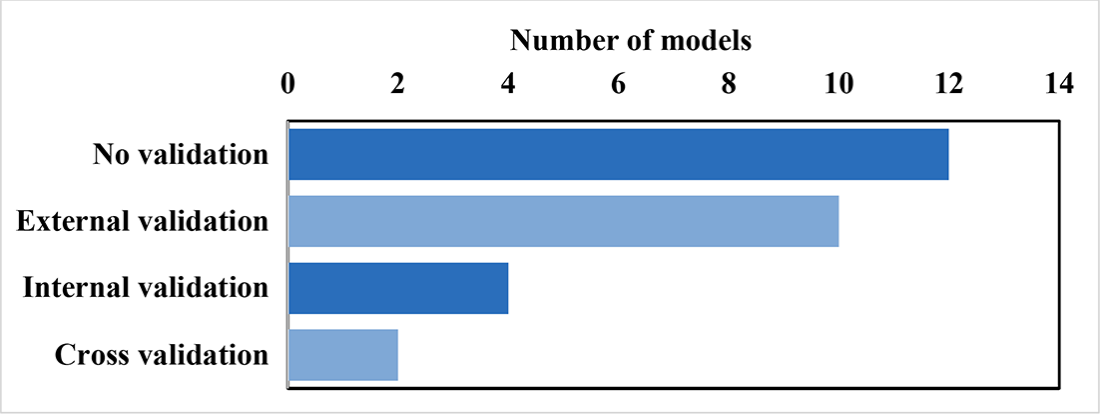
Occurrence of types of model validations (some models used more than one validation type).

Validation is used to check result accuracy. Figure 4 and online supplemental table S7 illustrate the wide gamut of validation approaches, including external validation, cross-validation and internal validation. We treated all models published in peer-reviewed journals as face validated by experts during the peer-review process. Hence, we did not count face validation for plotting or reporting.

External validation, considered the strongest validation form, was employed by eight models.^17 20 23–51 57 58 75–77 92 93 101–104 107–111^ BODE^3^ model and Cost-Effectiveness of Preventing AIDS Complications-US model employed cross-validations. However, some of the models did not mention the validation methods explicitly. Twelve (48%) models did not mention any validity check (without consideration of the face validation).^59–68 79–83 94–100 112–118^

Modellers perform a sensitivity analysis to check model outputs’ variation by input uncertainty.^106^ All models in our review reported some sensitivity analysis using one-way, multivariable or probabilistic sensitivity analysis (PSA). Three models applied a one-way sensitivity test only.^89–91 103 104 117^ Five models applied PSA only.^20 25 43–51 67 68 75–78 118^ Additionally, eight models used various approaches when testing different input parameters.^17 57–66 70–74 99–102 112–116^

#### Equity

Given the strong socioeconomic gradient of smoking in many countries, we also considered it essential to report policy outcomes on equity. Four models reported policy equity impact.^20 23 25 43–51 70–74 107–111 117^ A range of socioeconomic status (SES) measures were used. IMPACT model used area deprivation (index of relative area-level deprivation) or education level to indicate SES^23 107–111^; CDM defined SES by education levels^117^; BODE^3^ used ethnicity groups^20 25 43–51^ and ECEA tobacco tax model modelled income quintiles.^70–74^

### Developing a proof-of-concept quality assessment framework

Online supplemental table S5 shows the models scored using the proposed quality assessment framework and presents the number of published articles using the model. BODE^3^ was the highest scored model with one missing point on using the categorical smoking status. The models with higher quality scores were generally associated with more peer-reviewed publications (online supplemental figure S1 and online supplemental table S8).

## Discussion

This tobacco control PSM systematic review critically analysed existing models’ strengths and weaknesses regarding data inputs, model structure and outputs. Going beyond previous systematic reviews, we then devised and proposed a tobacco control PSM quality assessment framework. This quality framework could potentially be used in future research to enable readers to better assess the quality of tobacco control PSMs.

Our systematic review confirmed the multitude of modelling techniques used in the field. It revealed a wide range of quality, with few achieving high scores. The diffusion of good modelling practices thus currently appears to be suboptimal.

All included models had been subjected to sensitivity analysis and most appropriately used population data from public or administrative registries to represent the population.^106 119^ However, other best practices were often lacking.

Few models adequately captured the epidemiology of smoking harms. Smoking intensity and duration are essential,^120^ and the risk from smoking is cumulative, especially for cancers and COPD. The risk reduction after smoking cessation is likewise gradual. In addition, considerable time lags between exposure and change in risk exist for some diseases. By ignoring these factors, many models risk overestimating the impact of the simulated tobacco control policies.^121^

Furthermore, around one in five models used no empirical evidence to inform policy effectiveness. Thus, risking substantial bias.

Most models provided documentation to explain technical details for readers. However, none offered the source code under an open-source licence to enable complete transparency and scrutiny. We consider this a missed opportunity for transparency and sharing good practice, avoiding unnecessary repetition of work between research groups, and enabling more rapid model development.^14 106 122 123^ Ultimately, these coding silos hinder evidenced-based health policymaking and evaluation by needlessly slowing down model development and the dispersion of good practice.

Only four models reported on the potential equity of the simulated tobacco control policies. This is despite smoking prevalence having strong socioeconomic gradients in most countries; gradients which inequitable tobacco control policies have sometimes intensified.^121 124^

### Quality assessment framework

In developing our proof-of-concept quality assessment framework, we included nine dimensions. Each appeared feasible, being achieved in at least one tobacco control PSM. Reassuringly, the models with the highest quality scores were broadly those with a higher number of publications, although two high-quality models with high publication count perhaps drove the pattern.

### Public health implications

Policymakers could use this review as a registry of the currently available models. Furthermore, we propose an easy-to-use framework to assess the quality of the existing and future models, guide narratives of quality assessment during the peer-review process and foster progressively higher quality models.

Earlier guidelines powerfully informed our proposed quality assessment framework.^123^ However, we would suggest that most such guidelines are primarily useful for modellers rather than model users. They are lengthy (span across seven papers), challenging for non-technical users to digest and practice, and too generic to directly cover specific tobacco epidemiology characteristics (such as the cumulative nature of the risk and long lags between exposure and some diseases). These shortcomings may perhaps help explain the lack of any quality assessment in the two previous systematic reviews on tobacco models. We believe that our proposed quality assessment framework would be simple to apply directly to tobacco control PSMs and would not require the user to have any deep technical background.

The quality assessment framework we are proposing may also incentivise modelling complexity. We argue that complexity is necessary to integrate the increasingly available information, enabling richer, more accurate and comparable modelling outputs for policymakers and planners.^124^ Increased collaboration between modelling teams is thus urgently needed to mitigate many of the potential pitfalls of complexity and improve quality.

Organisations that facilitate collaboration among health policy modellers could play an important role. For instance, CISNET, a National Cancer Institute-funded modelling consortium, shares model common inputs and common intermediate/final outputs; modellers can then compare prediction results between different models.^125 126^

In the longer term, such collaboration would create a virtuous circle of modellers having a framework to support them and policymakers having consistently better models.

### Strengths and limitations

Building on previous reviews, we applied broader inclusion requirements and enhanced systematic methods. Our systematic review thus offers a broader and deeper view of the current tobacco control PSM landscape.

Additionally, we went beyond the traditional methodology review to provide an easy-to-use framework for the quality assessment of existing and future models. This should facilitate the development of higher quality tobacco control PSMs and may be useful during the peer-review process.

This review has potential limitations. First, we only analysed PSMs used in more than two studies to better represent the most actively used tobacco control PSMs. Likewise, we excluded models with more than two studies if these were all published before July 2013, as these can be found in the previous review by Feirman *et al*. Unavoidably, our approach has excluded some tobacco control PSMs. However, given the aims of our study, we would not expect them to have fundamentally different modelling practices than the models we included.

Second, allocating a single point in each of the nine (binary) dimensions of the quality framework was intended to be simple but risks being simplistic. However, in future real-world uses of the framework, we expect to refine these methods into more elaborate and weighted scoring schemes, perhaps tailored to the specific research aims. That further development and validation might permit an even broader and more robust assessment of model quality.

Third, due to resource constraints, we did not search for any grey literature or reports and only included studies published in English; we may thus have excluded some potentially influential models.

Finally, we included five studies funded by industry, which is liable to COI and bias. That represents a topic for further research.

## Conclusions

In conclusion, we have usefully highlighted the strengths and weaknesses of tobacco control PSMs’ data selection, model structure and output. We offer a nine-dimension proof-of-concept quality assessment framework to help facilitate the development of high-quality policy models in tobacco control, and perhaps more widely.

**What this paper adds**

**What is already known on this subject**

- Tobacco control policy simulation models have been used to guide tobacco control policymaking during the planning stage and the evaluation of post-implementation impact. However, despite this being a very active research area, there is no widely accepted quality assessment framework for tobacco control policy simulation models.

What this paper adds

- Analysing the methodology of published tobacco control policy simulation models potentially offers a broader and deeper view of the current policy modelling landscape.
- We offer a proof-of-concept quality assessment framework for tobacco control policy simulation models, which may guide quality assessment narratives in the peer-review process and foster higher modelling standards.

## Data Availability

All data relevant to the study are included in the article or uploaded as supplemental information.
