# Supplementary material for: Identifying best modelling practices for tobacco control policy simulations: a systematic review and a novel quality assessment framework
Source: Tob Control. 2022 Jan 11;32(5):589–98. doi: 10.1136/tobaccocontrol-2021-056825 (PMC10447402; doi:10.1136/tobaccocontrol-2021-056825)
Supplement: Supplementary data [file tobaccocontrol-2021-056825supp001.pdf]

Identifying best modelling practices for tobacco control policy simulations: a systematic review and a novel quality assessment framework- Supplementary Materials

Vincy Huang, Anna Head, Lirije Hyseni, Martin O'Flaherty, Iain Buchan, Simon Capewell, Chris Kypridemos

Page	Content
2	Text S1. Search strategy for Tobacco control policy simulation models systematic review
6	Text S2. Potential good modelling practices
7	Text S3. Summary of included models
14	Text S4. List of papers not included in this systematic review
20	Table S1. Synthesis Without Meta-analysis (SWiM) reporting items
23	Table S2. PICOS: inclusion / exclusion criteria
24	Table S3. Data extraction form
29	Table S4. Occurrence of model outcome types
30	Table S5. Occurrence of number of disease groups simulated by models
31	Table S6. Diseases groups included in models
32	Table S7. Occurrence of model validations types
33	Table S8. Model score
35	Figure S1. Model score and number of peer-reviewed publications linked to the model

Text S1. Search strategy (adapted from Feirman et al., 2016).

PubMed

(("models, theoretical"[majr:noexp] OR "models, statistical"[majr:noexp] OR "models, economic"[majr] OR "computer simulation"[majr:noexp] OR "monte carlo method"[mesh] OR "decision support techniques"[majr:noexp] OR "decision trees"[mesh] OR "systems theory"[mesh] OR "markov chains"[mesh] OR "system dynamics"[tiab] OR "agent-based model"[tiab] OR "agent-based models"[tiab] OR "agent-based modeling"[tiab] OR "agent-based modelling"[tiab] OR "simulation model"[tiab] OR "decision analysis"[tiab] OR "decision framework"[tiab] OR "markov"[tiab] OR "cost-utility analysis"[tiab] OR "cost-utility analyses"[tiab] OR "cost-effectiveness analysis"[tiab] OR "cost-effectiveness analyses"[tiab] OR "cost-benefit analysis"[tiab] OR "cost-benefit analyses"[tiab] OR "forecasting"[mesh] OR "microsimulation"[tiab] OR "micro simulation"[tiab] OR "monte carlo"[tiab] OR "life year"[tiab] OR "life years"[tiab] OR "smoking-attributable deaths"[tiab] OR "smoking attributable deaths"[tiab] OR "deterministic"[tiab] OR "probabilistic"[tiab] OR "stochastic"[tiab] OR "dynamic transmission model"[tiab] OR "state-transition"[tiab] OR "state transition"[tiab] OR "discrete event"[tiab] OR "continuous event"[tiab] OR "analytic horizon"[tiab] OR "cohort simulation"[tiab] OR "second-order simulation"[tiab] OR "threshold analysis"[tiab] OR "years of healthy life"[tiab] OR "decision problem"[tiab] OR "transition probabilities"[tiab] OR "discount rate"[tiab]) AND ("Smoking"[Mesh] OR "Smoking Cessation"[Mesh] OR "Tobacco"[Mesh] OR "Tobacco Products"[Mesh] OR "Tobacco, Smokeless"[Mesh] OR "Smoking"[TI] OR "Tobacco"[TI] OR "Smoker"[TI] OR "Smokers"[TI] OR (cigar[TI] OR cigar'[TI] OR cigareftes[TI] OR cigaret[TI] OR cigarete[TI] OR cigarets[TI] OR cigarett[TI] OR cigarette[TI] OR cigarette'[TI] OR cigarette's[TI] OR cigarettedagger[TI] OR cigaretteinduced[TI] OR cigarettes[TI] OR cigarettes'[TI] OR cigarettesmoke[TI] OR cigaretts[TI] OR cigarillo[TI] OR cigarillos[TI] OR cigarlike[TI] OR cigarra[TI] OR cigarret[TI] OR cigarrette[TI] OR cigarrilla[TI] OR cigarro[TI] OR cigarros[TI] OR cigars[TI]) OR "Smokeless"[TIAB] OR (e cigarette[TIAB] OR e cigarette's[TIAB] OR e cigarettedagger[TIAB] OR e cigarettee[TIAB] OR e cigarettes[TIAB]) OR (electronic cigarette[TIAB] OR electronic cigarettes[TIAB]) OR "Snus"[TIAB] OR "Nicotine"[TIAB]))

CINAHL Plus

(MJ Computer Simulation OR Models, Statistical OR Forecasting OR Cost Benefit Analysis OR Quality-Adjusted Life Years OR TX “system dynamics” OR “agent-based model” OR “agent-based models” OR “agent-based modeling” OR “agent-based modelling” OR “simulation model” OR “decision analysis” OR “decision framework” or

"markov" OR "cost-utility analysis" OR "cost-utility analyses" OR "cost-effectiveness analysis" OR "cost-effectiveness analyses" OR "cost-benefit analysis" or "cost-benefit analyses" OR "microsimulation" OR "micro simulation" OR "monte carlo" OR "life year" OR "life years" OR "deterministic" OR "probabilistic" OR "stochastic" OR "dynamic transmission model" OR "state-transition" OR "state transition" OR "discrete event" OR "continuous event" OR "analytic horizon" OR "cohort simulation" OR "second-order simulation" OR "first-order simulation" OR "threshold analysis" OR "years of healthy life" OR "decision problem" OR "transition probabilities" OR "discount rate") AND (MJ Tobacco OR Smoking OR Smoking Cessation OR Smoking—Trends OR Smoking Cessation OR TX smokeless OR "Smoking" OR "Tobacco" OR "Smoker" or "Smokers" OR Cigar* OR "Smokeless" OR E-cigarette* OR Electronic cigarette* OR "Snus" OR "Nicotine" OR "smoking-attributable deaths" OR "smoking attributable deaths")

Limit: English Language

PsycINFO

((KW cost effectiveness OR economic analysis OR smoking-attributable deaths OR quality adjusted life expectancy OR economic impact OR SU "Costs and Cost Analysis" OR Health Care Policy OR Simulation OR Decision Making OR Life Expectancy OR TX "system dynamics" OR "agent-based model" OR "agent-based models" OR "agent-based modeling" OR "agent-based modelling" OR "simulation model" OR "decision analysis" OR "decision framework" or "markov" OR "cost-utility analysis" OR "cost-utility analyses" OR "cost-effectiveness analysis" OR "cost-effectiveness analyses" OR "cost-benefit analysis" or "cost-benefit analyses" OR "microsimulation" OR "micro simulation" OR "monte carlo" OR "life year" OR "life years" OR "deterministic" OR "probabilistic" OR "stochastic" OR "dynamic transmission model" OR "state-transition" OR "state transition" OR "discrete event" OR "continuous event" OR "analytic horizon" OR "cohort simulation" OR "second-order simulation" OR "first-order simulation" OR "threshold analysis" OR "years of healthy life" OR "decision problem" OR "transition probabilities" OR "discount rate") AND (KW tobacco control policies OR tobacco control policy OR smoking cessation OR smokeless tobacco OR cession treatment policies OR population smoking prevalence OR tobacco elimination OR cessation programs OR cigarette consumption OR smoking OR snus OR electronic cigarettes OR SU Smoking Cessation OR Tobacco Smoking OR Smokeless Tobacco OR TX smokeless OR "Smoking" OR "Tobacco" OR "Smoker" or "Smokers" OR Cigar* OR "Smokeless" OR E-cigarette* OR Electronic cigarette* OR "Snus" OR "Nicotine" OR "smoking-attributable deaths" OR "smoking attributable deaths"))

Population Group: Human

Language: English

Population: unselect animal

EMBASE

“theoretical model”/mj OR “statistical model”/mj OR “computer simulation”/mj OR “disease simulation”/mj OR “monte carlo method”/mj OR “decision support system”/mj OR “decision tree”/mj OR “systems theory”/mj OR “economic evaluation”/exp OR “forecasting”/exp OR “economic model”:ab,ti OR “simulation model”:ab,ti OR “markov”:ab,ti OR “systems dynamics”:ab,ti OR “agent-based model”:ab,ti OR “agent-based models”:ab,ti OR “agent-based modeling”:ab,ti OR “agent-based modelling”:ab,ti OR “decision analysis”:ab,ti OR “decision framework”:ab,ti OR “microsimulation”:ab,ti OR “micro simulation”:ab,ti OR “life year”:ab,ti OR “life years”:ab,ti OR “smoking-attributable deaths”:ab,ti OR “smoking attributable deaths”:ab,ti OR “deterministic”:ab,ti OR “probabilistic”:ab,ti OR “stochastic”:ab,ti OR “dynamic transmission model”:ab,ti OR “state-transition”:ab,ti OR “state transition”:ab,ti OR “discrete event”:ab,ti OR “continuous event”:ab,ti OR “analytic horizon”:ab, ti OR “cohort simulation”:ab,ti OR “second-order simulation”:ab,ti OR “first-order simulation”:ab,ti OR “threshold analysis”:ab,ti OR “years of healthy life”:ab,ti OR “decision problem”:ab,ti OR “transition probabilities”:ab,ti OR “discount rate”:ab,ti

AND

‘smoking’/mj OR ‘cigarette smoke’/mj OR ‘bidi smoking’/mj OR ‘smoking regulation’ OR ‘smoking cessation’/exp OR ‘tobacco’/exp OR ‘smokeless tobacco’/exp OR ‘electronic cigarette’:ab,ti OR ‘e-cigarette’:ab,ti OR ‘snus’: ab,ti OR ‘nicotine’:ab,ti

NOT ‘cannabis smoking’/exp NOT ‘cigarette smoke condensate’/mj

EconLit

CC I180 OR CC C530 OR CC J110 OR KW “Simulation” OR CC I120 OR TX “system dynamics” OR “agent-based model” OR “agent-based models” OR “agent-based modeling” OR “agent-based modelling” OR “simulation model” OR “decision analysis” OR “decision framework” or “markov” OR “cost-utility analysis” OR “cost-utility analyses” OR “cost-effectiveness analysis” OR “cost-effectiveness analyses” OR “cost-benefit analysis” or “cost-benefit analyses” OR “microsimulation” OR “micro simulation” OR “monte carlo” OR “life year” OR “life years” OR “deterministic” OR “probabilistic” OR “stochastic” OR “dynamic transmission model” OR “state-transition” OR

"state transition" OR "discrete event" OR "continuous event" OR "analytic horizon" OR "cohort simulation" OR "second-order simulation" OR "first-order simulation" OR "threshold analysis" OR "years of healthy life" OR "decision problem" OR "transition probabilities" OR "discount rate"

AND

KW "Smoking" OR "tobacco" OR TX smokeless OR "Smoking" OR "Tobacco" OR "Smoker" or "Smokers" OR Cigar* OR "Smokeless" OR E-cigarette* OR "Electronic cigarette*" OR "Snus" OR "Nicotine" OR "smoking-attributable deaths" OR "smoking attributable deaths"

Filter: only English

Text S2. Potential Good Modelling Practices.

We examined the modelling approaches by a) model inputs (hierarchy of evidence, population representativeness), b) model structure (exposure granularity, disease epidemiology, documentation), and c) model outputs (reporting standards, uncertainty and sensitivity analysis, model validation) to identify method strengths and weaknesses.

Text S3. Summary of included models (in descending order of the number of peer-reviewed articles).

SimSmoke

SimSmoke is a first-order Markov model to estimate the smoking prevalence changes and smoking-attributable deaths of various tobacco control policies. SimSmoke relies on four sub-modules – population size, smoking prevalence, smoking-attributable deaths, and policy modules. Risk factors included categorical smoking status and the year since quitting. Model outcomes focus on mortality and smoking prevalence. SimSmoke was calibrated, and sensitivity analysis was performed. Readers are provided with the model documentation. The model was reported with external validation. However, there were no simulated diseases mentioned in the model. SimSmoke was also used to model smoking behaviour by dual users (SLT and cigarettes or snus and cigarettes).

Abridged SimSmoke

Abridged SimSmoke is a model that uses a single year to project policy short-term (5 years), mid-term (15 years), and long-term (40 years) effects on smoking prevalence and smoking-attributable deaths. Slightly different from the four modules in SimSmoke, Abridged SimSmoke utilises three components population size, smoking prevalence and policy modules in the approach. In this model, populations are stratified with an unemployed status.

BODE^3

BODE^3 is a multistate life-table model of 16 smoking-related diseases. It was developed to evaluate intervention effectiveness in reducing smoking prevalence, related diseases, cost, cost-effectiveness and equity on ethnicity groups. Model result certainty was reported. However, the model only modelled policy impact on New Zealand populations. There were 16 diseases included in the model - chronic obstructive pulmonary disease (COPD), cardiovascular disease (CVD), stroke, lung cancer. Probabilistic sensitivity analysis (PSA). Moreover, cross-validation and external validation were performed for this model. This model includes two modules: a population forecasting model and a multiple-state life-table.

Extended cost-effectiveness analysis (ECEA) tobacco tax model

The extended cost-effectiveness analysis (ECEA) tobacco tax model is a cost-effectiveness model in estimating the impact of tobacco taxation. It was adapted from the Asian Development Bank's framework. The population groups were stratified by income quintile. It included diseases such as COPD, CVD, stroke, lung cancer, bladder cancer and

neoplasms. The model generates cost, mortality, the number of smokers who quit, life-years gained, additional revenues generated and equity outcomes.

Moreover, it was tested with one-way sensitivity analysis and validated. The model technical document is available for readers. Nevertheless, the majority of the studies using this model focused on male-only.

IMPACT

IMPACT is a cell-based model to estimate CHD mortality changes under different policy scenarios. Risk factors included blood pressure, cholesterol, diabetes, fruit and vegetable, smoking (never smoker, long-term ex-smoker, recent ex-smoker, current smoker), salt intake, saturated fat intake, BMI and physical activities. Model simulated diseases include CHD and type 2 Diabetes. In the IMPACT model, population characteristics include age, gender and socioeconomics classes (indicated by QIMD). The model projects outcomes on equity, CHD mortality, smoking prevalence and life-years gained. Moreover, the resulting uncertainty was reported. Probabilistic sensitivity analysis (PSA) using the Monte Carlo approach was applied as the sensitivity analysis, and the model was externally validated. Moreover, the model documentation is available to readers.

European study on Quantifying Utility of Investment in Protection from Tobacco model (EQUIPTMOD)

The European study on Quantifying Utility of Investment in Protection from Tobacco model (EQUIPTMOD) is constructed as a Markov state transition model. It models smoking cessation on four diseases: stroke, lung cancer, coronary heart disease and COPD. It provides economic estimates on intervention cost, return on investment (ROI), incremental cost-effectiveness ratio (ICER) and quality-adjusted life-year (QALY). Both univariable sensitivity analysis and PSA were performed. Technical document for all countries is available on the study website. However, there was no model validity mentioned in the papers.

Benefits of Smoking Cessation on Outcomes (BENESCO) model

Benefits of Smoking Cessation on Outcomes (BENESCO) model is a discrete-time Markov model that estimates the cost-effectiveness of a single smoking cessation attempt. Smokers were modelled by quit smoking duration, including smoker, recent quitter and long-term quitter. COPD, CHD, stroke and lung cancer were included in the model. Results on mortality, morbidity, cost and QALY were generated. In addition, univariable sensitivity analysis and PSA was performed on this model. It was calibrated. However, there is no documentation provided. In addition, funding was provided by Pfizer.

Two-quit BENESCO is a model developed based on the adaption of BENESCO to model smokers that attempts two times quit smoking over a lifetime. Diseases include COPD, CHD, stroke and asthma exacerbations were modelled. One-way and PSA were performed for this model. Scenario testing and face validation were applied for this model.

DYNAMO-HIA model

The DYNAMO-HIA is a software applying a discrete-time, Markov-type multistate model. The model combines a microsimulation to simulate the risk factor exposure development and projecting the health impact over time with a macrosimulation. Moreover, three modules - population, disease, risk factors were included; eight health risk factors were included - BMI, alcohol, smoking, second-hand smoking, salt intake, physical activities, obesity. The model simulates nine smoking-related diseases: ischemic heart diseases (IHD), diabetes, COPD, stroke, lung, breast, colorectal, oral, and oesophageal cancer. The model estimates the chances of morbidity and healthy life years (HLY). The model validity checked was mention for this model.

Johansson model

Johansson model is a Markov-cycle tree model. It simulates smoking cessation on COPD, cardiovascular disease (stroke and CHD) and cancers to estimate QALY and cost impact. Sensitivity analysis was performed using multivariable analysis and PSA. Model external validation was mentioned. Moreover, the model non-technical document is available.

Prevention Impacts Simulation Model (PRISM)

Prevention Impacts Simulation Model (PRISM) is an interactive system dynamics model for cardiovascular disease prediction. Users could interact with the model parameters using the user interface. It was designed to estimate policy impact on mortality, morbidity, healthcare cost, productivity and result uncertainty. A series of risk factors were included: blood pressure, cholesterol, second-hand smoking, obesity, psychological distress, fruit and vegetable, smoking (never smoker, long-term ex-smoker, recent ex-smoker, current smoker), blood glucose categories, periodontal disease, sleep apnoea, small particulate air pollution, and inadequate use of aspirin for primary prevention. The model was externally validated, and the sensitivity analysis was checked with PSA. However, it was only applied to the US setting.

Jiménez model

Jiménez et al. developed the budgetary impact analysis (BIA) model for the Spanish population. This model incorporates a hybrid model - closed cohort and Markov chains. The model population are represented by patients diagnosed with COPD, t2-DM and CVD, who would be willing to stop smoking. Risk factors included smoking status and willingness and quit history. The model estimates costs and the number of quitters. This model was internally validated and tested with univariable sensitivity analysis. Furthermore, this model received funding from Pfizer Inc.

Baker model

Baker et al. developed a closed cohort budget impact Markov model. The model estimates the cost of smoking cessation prescriptions from the angle of US payers. Categorical smoking status is the risk factor input. It predicts the number of quitters and medical expenditures under different policy scenarios. A series of univariate and multivariate sensitivity analyses were performed on the model. However, there was no mentioning of modelled diseases and no reporting of model validation. Moreover, the model documentation was not provided by modellers. From the declaration, the authors mentioned that IQVIA employees developed the model with funding from Pfizer.

Barnett model

Barnett model is a Markov model that used for smoking cessation trial cost-effectiveness. Treatment effectiveness is extracted from the trial. It predicts the trial lifetime effect on cost, mortality and QALYs. The result range is provided. This model was tested with a one-way sensitivity method. Its technical appendix is provided, but the code is not open-source. The model was calibrated; however, there was no mentioning of the model validation and no specific modelling of diseases mentioned for this model.

Cantor model

The model designed by Cantor et al. is a two-structured decision-analytic model to assess the cost-effectiveness of smoking cessation interventions over a lifetime. The first model evaluates cost per successful quit while the second one estimates life expectancy and quality-adjusted life expectancy. This model includes a lifetime horizon to capture the smoking intervention for long-term benefit—however, the model only simulated interventions in the United States. One-way and two-way sensitivity analysis were used. The model validation is not mentioned, and there is no additional model documentation provided.

Chevreul model

Chevreul model is a Markov state-transition model that is used to predict cost-effectiveness analysis of smoking policies on the French population. The model simulates the natural history of smokers until death. It only modelled smokers diagnosed with either lung cancer, COPD or CVD, such as stroke or coronary artery disease and death. Diseases include COPD, CVD and lung cancer. Moreover, health outcomes and ICER are provided by the model. The model used sensitivity tests and was cross-validated. The model documentation is available.

Cost-Effectiveness of Preventing AIDS Complications (CEPAC)-US model

Cost-Effectiveness of Preventing AIDS Complications (CEPAC)-US model is a microsimulation model of HIV natural history and treatment. It is applicable for the HIV-infected US population. The model includes risk factors - smoking intensity(packs/day), CD4+ T-cell count, viral load, history of the opportunistic disease, and antiretroviral therapy use. Lung cancer is simulated as a disease outcome. The model predicts the number of years of life lost from smoking. Two-way sensitivity analysis was applied in this model. Moreover, this model was validated with internal and external validation. There is a link to model documentation provided; however, it is not open access.

ModelHeath: Tobacco

ModelHeath: Tobacco is a microsimulation model developed by Maciosek et al. ModelHealth: Tobacco MN is the same model for modelling the population data from Minnesota. Detailed demographic information including education level, ethnicity, disability, employment and poverty were modelled. Disease including CVD, stroke, lung cancer and respiratory disease was simulated. The model reports the health burden and cost-effectiveness of smoking behaviour, including medical cost, hospitalisation, mortality and morbidity, productivity loss, QALY and smoking prevalence. One-way sensitivity analysis was performed. Moreover, the model was validated with internal and external validation. Model documentation is provided for the readers.

Parrott model

Parrott model is used in evaluating the cost-effectiveness of clinical trials over a lifetime. The policy effectiveness was extracted from a randomised controlled trial, and other data inputs were either from the trial or national representative surveys. Diseases including COPD, CHD, stroke, lung cancer, asthma, pregnancy-related (placental abruption, ectopic pregnancy, pre-eclampsia, placenta previa and miscarriage infant morbidities: low infant birth weight, stillbirth, premature birth) were modelled. The model estimates trial outcomes on cost and QALY with a result uncertainty

range. The result was tested with PSA. Users are provided with model documentation. However, there was no mentioning of model validation.

Population Health Impact Model (PHIM)

Population Health Impact Model (PHIM) is a tobacco industry funded model by Philip Morris International. This model evaluates the health impact of a candidate modified risk tobacco product (cMRTP). It projects cMRTP uptake and mortality rate changes under alternative scenarios. cMRTP users and dual users were counted as the smoking status. In addition, smoking-related attributable deaths from lung cancer, ischemic heart disease, stroke and chronic obstructive pulmonary disease were considered. This model was tested with sensitivity analysis and validated. PHIM model comprises two modules - a population module that generates distributions of smoking histories for each scenario at the end of the period being studied and an epidemiologic risk module to estimate smoking-related attributable deaths.

Tobacco Town

This is an agent-based model. Smoking intensity (cigarettes/day) is simulated in the model. Population characteristics include priority population representation(lesbian, LGBTQ+), income, urban rich, urban poor, suburban rich, suburban poor, mode of transport, home and work locations, and route between the two locations and ethnicities. The model predicts cost and tobacco purchase behaviour. The model reported calibration and sensitivity analysis. Moreover, there is additional model documentation provided. However, there was no mentioning of model validation.

UK Health Forum (UKHF) simulation

UK Health Forum (UKHF) simulation is a two structure microsimulation model to predict the health and economic impact of smoking policies within the UK setting. Module one applies a regression model to project smoking prevalence over time. Module two uses the smoking prevalence projection in a microsimulation model to estimate the cost and health benefits of policy scenarios. Seventeen smoking-related diseases (COPD, CHD, stroke, 14 tobacco-related cancers) were included in the model. The model generates outcomes on cost, morbidity and smoking prevalence. The model was tested with sensitivity analysis. There is detailed model documentation with equations. However, there was no mentioning of validation.

Chronic Disease Model (CDM)

Chronic Disease Model (CDM) is a dynamic multistate Markov model. This model simulated 20 chronic diseases. It models population groups stratified by age, gender and socioeconomics status using education levels. This model generates the lifetime outputs on QALYs, number of quitters and cost introduced by different smoking policies.

Coronary Heart Disease (CHD) Policy Model

Coronary Heart Disease (CHD) Policy Model is a state-transition Markov model that predicts policy impact on CHD incidence, prevalence, mortality and costs. This model includes three sub-models: demographic–epidemiological, bridge and disease-history. Six risk factors linking with CHD and stroke were simulated in this model. Moreover, this model was calibrated, and sensitivity analysis was performed.

Lung Cancer Policy Model (LCPM)

The Lung Cancer Policy Model is a state-transition microsimulation that models lung cancer development, screening and treatment at the individual patient level. Detailed patient smoking histories were counted in this model. This model was calibrated and validated.

Mendez model

Mendez model is an excel-based state-transition model. It composes two submodules, namely, prevalence and epidemiological models. The model generates outputs on smoking prevalence, health and cost-effectiveness under different tobacco interventions. This model only simulates the US population.

Mejia model

Mejia model used a decision tree model in Monte Carlo simulations. It estimates the health effects of expanding e-cigarette sales in the United States and the United Kingdom. Outcomes include smoking prevalence and costs with the uncertainty range provided. Sensitivity analysis was performed. There was no mentioning of any model validation.

Text S4. Models retrieved from the search cretiria that appeared only in one publication.

1. Altman D., Clement F.M., Barnieh L., *et al.* Cost-effectiveness of universally funding smoking cessation pharmacotherapy. *Can J Respir Crit Care Sleep Med* 2019;**3**:67–75. doi:10/ggm8qb
2. Ansah JP, Inn RLH, Ahmad S. An evaluation of the impact of aggressive hypertension, diabetes and smoking cessation management on CVD outcomes at the population level: a dynamic simulation analysis. *BMC Public Health* 2019;**19**:1105. doi:10/ggm9w5
3. Apelberg BJ, Feirman SP, Salazar E, *et al.* Potential Public Health Effects of Reducing Nicotine Levels in Cigarettes in the United States. *New England Journal of Medicine* 2018;**378**:1725–33. doi:10/cmmv
4. Aungkulanon S, Pitayarangsarit S, Bundhamcharoen K, *et al.* Smoking prevalence and attributable deaths in Thailand: predicting outcomes of different tobacco control interventions. *BMC Public Health* 2019;**19**:984. doi:10/ggm9ww
5. Bachand AM, Sulsky SI. A dynamic population model for estimating all-cause mortality due to lifetime exposure history. *Regulatory Toxicology and Pharmacology* 2013;**67**:246–51. doi:10/f5gz5x
6. Basu S, Sussman JB, Rigdon J, *et al.* Benefit and harm of intensive blood pressure treatment: Derivation and validation of risk models using data from the SPRINT and ACCORD trials. *PLoS Medicine* 2017;**14**:1–26. doi:10/gb4j99
7. Benmarhnia T, Dionne P-A, Tchouaket É, *et al.* Investing in a healthy lifestyle strategy: is it worth it? *Int J Public Health* 2017;**62**:3–13. doi:10/f9tf85
8. Bertram MY, Sweeny K, Lauer JA, *et al.* Investing in non-communicable diseases: an estimation of the return on investment for prevention and treatment services. *The Lancet* 2018;**391**:2071–8. doi:10.1016/S0140-6736(18)30665-2
9. Cedillo S, Sicras-Mainar A, Jimenez-Ruiz CA, *et al.* Budgetary Impact Analysis of Reimbursement Varenicline for the Smoking-Cessation Treatment in Patients with Cardiovascular Diseases, Chronic Obstructive Pulmonary Disease or Type-2 Diabetes Mellitus: A National Health System Perspective. *Eur Addict Res* 2017;**23**:7–18. doi:10/f9q5hw

10. Chao D, Hashimoto H, Kondo N. Social influence of e-cigarette smoking prevalence on smoking behaviours among high-school teenagers: Microsimulation experiments. *PLoS One* 2019;**14**:e0221557. doi:10/ggm9xc
11. Cherng ST, Tam J, Christine PJ, *et al.* Modeling the Effects of E-cigarettes on Smoking Behavior: Implications for Future Adult Smoking Prevalence. *Epidemiology* 2016;**27**:819–26. doi:10/f9g8vn
12. Connolly MP, Kotsopoulos N, Postma MJ, *et al.* The Fiscal Consequences Attributed to Changes in Morbidity and Mortality Linked to Investments in Health Care: A Government Perspective Analytic Framework. *Value Health* 2017;**20**:273–7. doi:10/gf3mh2
13. Djalalov S, Masucci L, Isaranuwatchai W, *et al.* Economic evaluation of smoking cessation in Ontario's regional cancer programs. *Cancer Med* 2018;**7**:4765–72. doi:10/gdvtdr
14. Getsios D. et al. Smoking Cessation Treatment and Outcomes Patterns Simulation: A New Framework for Evaluating the Potential Health and Economic Impact of Smoking Cessation Interventions | Kopernio. https://kopernio.com/viewer?doi=10.1007%2Fs40273-013-0070-5&token=WzIxMDU2NzcsIjEwLjEwMDcvczQwMjczLTAxMy0wMDcwLTUiXQ.AyKvwsUkIYn0SyFzGypATSyxico (accessed 22 Jun 2020).
15. Golden SD, Farrelly MC, Luke DA, *et al.* Comparing projected impacts of cigarette floor price and excise tax policies on socioeconomic disparities in smoking. *Tobacco Control* 2016;**25**:i60–6. doi:10/f89qcb
16. Goodchild M, Perucic A-M, Nargis N. Modelling the impact of raising tobacco taxes on public health and finance. *Bull World Health Organ* 2016;**94**:250–7. doi:10/f8nx8m
17. Grace RC, Kivell BM, Laugesen M. Predicting decreases in smoking with a cigarette purchase task: evidence from an excise tax rise in New Zealand. *Tob Control* 2015;**24**:582–7. doi:10/gg5mv9
18. Healey A, Roberts S, Sevdalis N, *et al.* A Cost-Effectiveness Analysis of Stop Smoking Interventions in Substance-Use Disorder Populations. *Nicotine Tob Res* 2019;**21**:623–30. doi:10/ggm74h
19. Hill A, Camacho OM. A system dynamics modelling approach to assess the impact of launching a new nicotine product on population health outcomes. *Regulatory Toxicology and Pharmacology* 2017;**86**:265–78. doi:10/ggm62g

20. Holford TR, Meza R, Warner KE, *et al.* Tobacco Control and the Reduction in Smoking-Related Premature Deaths in the United States, 1964-2012. *JAMA* 2014;**311**:164–71. doi:10/f5np3w
21. Igarashi A, Goto R, Suwa K, *et al.* Cost-Effectiveness Analysis of Smoking Cessation Interventions in Japan Using a Discrete-Event Simulation. *Appl Health Econ Health Policy* 2016;**14**:77–87. doi:10/f8rfb4
22. Islek D, Sozmen K, Unal B, *et al.* Estimating the potential contribution of stroke treatments and preventative policies to reduce the stroke and ischemic heart disease mortality in Turkey up to 2032: a modelling study. *BMC Public Health* 2016;**16**:46. doi:10/f77z9z
23. Jones M, Smith M, Lewis S, *et al.* A dynamic, modifiable model for estimating cost-effectiveness of smoking cessation interventions in pregnancy: application to an RCT of self-help delivered by text message. *Addiction* 2019;**114**:353–65. doi:10.1111/add.14476
24. Kontis V, Mathers CD, Rehm J, *et al.* Contribution of six risk factors to achieving the 25×25 non-communicable disease mortality reduction target: a modelling study. *The Lancet* 2014;**384**:427–37. doi:10.1016/S0140-6736(14)60616-4
25. Kowada A. Cost-effectiveness of tobacco cessation support combined with tuberculosis screening among contacts who smoke. ;:8. doi:10/f7f98r
26. Kuklinski MR, Fagan AA, Hawkins JD, *et al.* Benefit–cost analysis of a randomized evaluation of Communities That Care: Monetizing intervention effects on the initiation of delinquency and substance use through grade 12. *Journal of Experimental Criminology* 2015;**11**:165–92. doi:10/f7dptp
27. Kulaylat AS, Hollenbeak CS, Soybel DI. Cost-utility analysis of smoking cessation to prevent operative complications following elective abdominal colon surgery. *Am J Surg* 2018;**216**:1082–9. doi:10/ggm64g
28. Lal A, Mihalopoulos C, Wallace A, *et al.* The cost-effectiveness of call-back counselling for smoking cessation. *Tob Control* 2014;**23**:437–42. doi:10/f6rdwn
29. Levy DT, Borland R, Lindblom EN, *et al.* Potential deaths averted in USA by replacing cigarettes with e-cigarettes. *Tobacco Control* 2018;**27**:18–25. doi:10/gbzrmj

30. Levy DT, Yuan Z, Li Y, *et al.* A modeling approach to gauging the effects of nicotine vaping product use on cessation from cigarettes: what do we know, what do we need to know? *Addiction* 2019;**114 Suppl 1**:86–96. doi:10.1111/add.14530

31. Li Y, Kong N, Lawley M, *et al.* Advancing the use of evidence-based decision-making in local health departments with systems science methodologies. *Am J Public Health* 2015;**105 Suppl 2**:S217-222. doi:10.2105/AJPH.2014.302077

32. Luo L., Pang B., Chen J., *et al.* Assessing the impact of lifestyle interventions on diabetes prevention in China: A modeling approach. *Int J Environ Res Public Health* 2019;**16**. doi:10/ggm8qg

33. Marynak KL, Xu X, Wang X, *et al.* Estimating the Impact of Raising Prices and Eliminating Discounts on Cigarette Smoking Prevalence in the United States. *Public Health Rep* 2016;**131**:536–43. doi:10.1177/0033354916662211

34. McAuley A, Denny C, Taulbut M, *et al.* Informing Investment to Reduce Inequalities: A Modelling Approach. *PLOS ONE* 2016;**11**:e0159256. doi:10.1371/journal.pone.0159256

35. McCallum DM, Fosson GH, Pisu M. Making the Case for Medicaid Funding of Smoking Cessation Treatment Programs: An Application to State-Level Health Care Savings. *Journal of Health Care for the Poor & Underserved* 2014;**25**:1922–40. doi:10/f6sc45

36. Muhammad-Kah RS, Pithawalla YB, Boone EL, *et al.* A Computational Model for Assessing the Population Health Impact of Introducing a Modified Risk Claim on an Existing Smokeless Tobacco Product. *Int J Environ Res Public Health* 2019;**16**:E1264. doi:10.3390/ijerph16071264

37. Nemeth B, Kulchaitanaroaj P, Lester-George A, *et al.* A utility of model input uncertainty analysis in transferring tobacco control-related economic evidence to countries with scarce resources: results from the EQUIPT study. *Addiction* 2018;**113 Suppl 1**:42–51. doi:10/ggm63p

38. Ngalesoni F, Ruhago G, Mayige M, *et al.* Cost-effectiveness analysis of population-based tobacco control strategies in the prevention of cardiovascular diseases in Tanzania. *PLoS One* 2017;**12**:e0182113. doi:10/ggm622

39. Pennington B, Filby A, Owen L, *et al.* Smoking Cessation: A Comparison of Two Model Structures. *Pharmacoeconomics* 2018;**36**:1101–12. doi:10/ggm632

40. Pinto M, Bardach A, Palacios A, *et al.* Burden of smoking in Brazil and potential benefit of increasing taxes on cigarettes for the economy and for reducing morbidity and mortality. *Cadernos de Saúde Pública* 2019;**35**. doi:10/gg5mwb

41. Platt JM, Keyes KM, Galea S. Efficiency or equity? Simulating the impact of high-risk and population intervention strategies for the prevention of disease. *SSM Popul Health* 2017;**3**:1–8. doi:10.1016/j.ssmph.2016.11.002

42. Poland B. Population Modeling of Modified Risk Tobacco Products Accounting for Smoking Reduction and Gradual Transitions of Relative Risk. https://kopernio.com/viewer?doi=10.1093%2Fntr%2Fntx070&token=WzIxMDU2NzcsIjEwLjEwOTMvbnRyL250eDA3MCJd.IFnWlJvql5E8bC5l8Re68t4Pwxw (accessed 18 Apr 2020).

43. Popp J, Nyman JA, Luo X, *et al.* Cost-effectiveness of enhancing a Quit-and-Win smoking cessation program for college students. *Eur J Health Econ* 2018;**19**:1319–33. doi:10/ggm63z

44. Rana P, Perez-Rios M, Santiago-Perez MI, *et al.* Impact of a comprehensive law on the prevalence of tobacco consumption in Spain: evaluation of different scenarios. *Public Health* 2016;**138**:41–9. doi:10/ggm9xv

45. Sadatsafavi M, Ghanbarian S, Adibi A, *et al.* Development and Validation of the Evaluation Platform in COPD (EPIC): A Population-Based Outcomes Model of COPD for Canada. *Medical Decision Making* 2019;**39**:152–67. doi:10/gfvh6v

46. Saidi O., O'Flaherty M., Zoghlami N., *et al.* Comparing strategies to prevent stroke and ischemic heart disease in the tunisian population: Markov modeling approach using a comprehensive sensitivity analysis algorithm. *Comp Math Methods Med* 2019;**2019**. doi:10/gf654t

47. Saxena K. Development and validation of a discrete event simulation model to evaluate the long-term use of electronic cigarettes on the U.S. population. *J Manag Care Spec Pharm* 2016;**22**:S97.

48. Selya AS, Ivanov O, Bachman A, *et al.* Youth smoking and anti-smoking policies in North Dakota: a system dynamics simulation study. *Subst Abuse Treat Prev Policy* 2019;**14**:34. doi:10/ggm748

49. Shang C, Yadav A, Stoklosa M, *et al.* Country-specific costs of implementing the WHO FCTC tobacco control policies and potential financing sources. *PLOS ONE* 2018;**13**:e0204903. doi:10/gfcsbt

50. Sonntag D, Gilbody S, Winkler V, *et al.* German EstSmoke: estimating adult smoking-related costs and consequences of smoking cessation for Germany. *Addiction* 2018;**113**:125–36. doi:10/ggm75r
51. Sung H-Y, Penko J, Cummins SE, *et al.* Economic Impact of Financial Incentives and Mailing Nicotine Patches to Help Medicaid Smokers Quit Smoking: A Cost-Benefit Analysis. *Am J Prev Med* 2018;**55**:S148–58. doi:10.1016/j.amepre.2018.08.007
52. Tosanguan J, Chaiyakunapruk N. Cost-effectiveness analysis of clinical smoking cessation interventions in Thailand. *Addiction* 2016;**111**:340–50. doi:10/ggm9xf
53. Villanti AC, Jiang Y, Abrams DB, *et al.* A Cost-Utility Analysis of Lung Cancer Screening and the Additional Benefits of Incorporating Smoking Cessation Interventions. *PLoS One* 2013;**8**:e71379. doi:10/f5c83w
54. Warner KE, Mendez D. E-cigarettes: Comparing the Possible Risks of Increasing Smoking Initiation with the Potential Benefits of Increasing Smoking Cessation. *Nicotine & Tobacco Research* 2019;**21**:41–7. doi:10.1093/ntr/nty062
55. West R, Coyle K, Owen L, *et al.* Estimates of effectiveness and reach for 'return on investment' modelling of smoking cessation interventions using data from England. *Addiction* 2018;**113**:19–31. doi:10/gg2zwn
56. Xu X, Alexander RLJ, Simpson SA, *et al.* A cost-effectiveness analysis of the first federally funded antismoking campaign. *Am J Prev Med* 2015;**48**:318–25. doi:10/ggm94k
57. Yang W, Zou Q, Tan E, *et al.* Future Health and Economic Impact of Comprehensive Tobacco Control in DoD: A Microsimulation Approach. *Mil Med* 2018;**183**:e104–12. doi:10/gc4tm7
58. *La Foucade A, Gabriel S, Scott E, et al. Increased taxation on cigarettes in Grenada: potential effects on consumption and revenue. Rev Panam Salud Publica 2018;42:1–7. doi:10.26633/RPSP.2018.195*

Table S1. Synthesis Without Meta-analysis (SWiM) reporting items.

The citation for the Synthesis Without Meta-analysis explanation and elaboration article is: Campbell M, McKenzie JE, Sowden A, Katikireddi SV, Brennan SE, Ellis S, Hartmann-Boyce J, Ryan R, Shepperd S, Thomas J, Welch V, Thomson H. Synthesis without meta-analysis (SWiM) in systematic reviews: reporting guideline BMJ 2020;368:l6890

**SWiM is intended to complement and be used as an extension to PRISMA**			
**SWiM reporting item**	**Item description**	**Page in manuscript where item is reported**	**Other***
Methods			
**1** Grouping studies for synthesis	1a) Provide a description of, and rationale for, the groups used in the synthesis (e.g., groupings of populations, interventions, outcomes, study design)	Page 8 - 9	
	1b) Detail and provide rationale for any changes made subsequent to the protocol in the groups used in the synthesis	NA	
**2** Describe the standardised metric and transformation methods used	Describe the standardised metric for each outcome. Explain why the metric(s) was chosen, and describe any methods used to transform the intervention effects, as reported in the study, to the standardised metric, citing any methodological guidance consulted	NA	
**3** Describe the synthesis methods	Describe and justify the methods used to synthesise the effects for each outcome when it was not possible to undertake a meta-analysis of effect estimates	Page 8 - 9	

**4** Criteria used to prioritise results for summary and synthesis	Where applicable, provide the criteria used, with supporting justification, to select the particular studies, or a particular study, for the main synthesis or to draw conclusions from the synthesis (e.g., based on study design, risk of bias assessments, directness in relation to the review question)	Page 9	
**SWiM reporting item**	**Item description**	**Page in manuscript where item is reported**	**Other***
**5** Investigation of heterogeneity in reported effects	State the method(s) used to examine heterogeneity in reported effects when it was not possible to undertake a meta-analysis of effect estimates and its extensions to investigate heterogeneity	Page 9	
**6** Certainty of evidence	Describe the methods used to assess the certainty of the synthesis findings	NA	
**7** Data presentation methods	Describe the graphical and tabular methods used to present the effects (e.g., tables, forest plots, harvest plots).<br><br>Specify key study characteristics (e.g., study design, risk of bias) used to order the studies in the text and any tables or graphs, clearly referencing the studies included	Page 9 - 10	
Results			

**8** Reporting results	For each comparison and outcome, provide a description of the synthesised findings and the certainty of the findings. Describe the result in language that is consistent with the question the synthesis addresses, and indicate which studies contribute to the synthesis	Page 10 - 41	
Discussion			
**9** Limitations of the synthesis	Report the limitations of the synthesis methods used and/or the groupings used in the synthesis and how these affect the conclusions that can be drawn in relation to the original review question	Page 45 - 46	

PRISMA=Preferred Reporting Items for Systematic Reviews and Meta-Analyses.

*If the information is not provided in the systematic review, give details of where this information is available (e.g., protocol, other published papers (provide citation details), or website (provide the URL)).

Table S2. PICOS: inclusion / exclusion criteria.

Include	Exclude
**Participants**	
Studies on any human populations	Studies on animals and cells
**Interventions**	
Tobacco control policies	Non-tobacco control policies (e.g. cancer screening program)
**Comparator**	
Studies where tobacco control PSMs are evaluated or compared	No tobacco control PSMs presented
**Outcomes**	
Studies reporting any tobacco-related outcomes	Studies reporting no tobacco-related outcomes
**Study design**	
PSMs	Studies without PSMs

Table S3. Data extraction form.

Paper Name	Tick if yes
1. GENERAL INFORMATION	
Paper author (First author)	
Paper published year (published online)	
Ref ID (DOI):	
Data extractor:	
Extraction date (DD/MM/YYYY)	
Funding & Conflict of interest	
General information - Others	
2.MODEL DETAILS	
Model name	
Code license/ Open source	
code URL	
Model setting - Country/Area	
Model - Initial year	
Prediction period:	
model detail - others	
3.TYPE OF MODEL	
Agent-based model	
Decision tree	
Discrete event	
Life table	
Markov model	
Macrosimulation	
Microsimulation	
System dynamic	
Open cohort	
Close cohort:	
Continuous time	

Discrete-time	
Type of model - others	
4.DEMOGRAPHIC CHARACTERISTICS [illegible]	
Gender (Y, both, F, M)	
Age	
Socioeconomic status	
Education	
Income	
Race/ Ethnicity	
Urban/ Rural	
Demographic - Others	
5.RISK FACTORS	
Alcohol intake	
Alcohol intake (Unit)	
Blood pressure	
Blood pressure (Unit)	
Cholesterol	
Cholesterol (Unit)	
Competing causes	
Competing causes (Unit)	
Diabetes	
Diabetes (Unit)	
Environmental tobacco smoking	
Environmental tobacco smoking (Unit)	
Fruit and vegetable consumption	
Fruit and vegetable consumption (Unit)	
General Health status	
General Health status (Unit)	
Hypertension	
Hypertension (Unit)	

Mental health	
Mental health (Unit)	
Obesity or BMI	
Obesity or BMI (Unit)	
Physical activity	
Physical activity (Unit)	
Other risk factors (list down in box)	
Other risk factors (list down in box) (Unit)	
Smoking Status (never, former, smoker) (Unit)	
Smoking status (Unit)	
Smoking history (age star/ duration, intensity/age quit)	
Unit (pack-year, smoking duration, smoking intensity, smoking duration and intensity [illegible]	
Lag time	
Lag time (Unit)	
Risk factor-others	
Risk factor-others (Unit)	
6.OUTCOME TYPE	
Equality	
Economics outcome	
Hospital admission	
Health outcomes - mortality	
Health outcomes - morbidity	
Health outcomes - other	
Smoking attitude/ Smoking prevalence	
Uncertainty	
Outcome types - Others (please describe)	
7.DISEASE CATEGORIES	
AMI (Acute myocardial infarction)	
Atrial fibrillation (AF)	
Asthma	

COPD	
CVD	
Diabetes	
Diabetic neuropathy	
Diabetic retinopathy	
Dyslipidaemia	
Lung cancer	
Obesity	
Other cancers	
Stroke	
Tuberculosis (TB)	
Hypertension	
Diseases - Others	
Disease categories - others	
8.DATA SOURCES USED	
Population	
Mortality	
Morbidity	
Policy effective/ treatment effectiveness	
Data source - Others	
9.MODEL CHECKING	
Any sensitivity analyses carried out?	
Which sensitivity analyses were carried out?	
Was the model aligned?	
Was the model calibrated?	
How was the model calibrated?	
Was the validity of the model tested?	
Face validation	
Internal validation	
Cross-validation	

External validation	
How was the validity quantified? *(e.g. % explained)*	
Validation - others	
Nontechnical & Technical documentation	
Assumptions	
Model availability for the reader (not including source code)	
Transparency - others	
Model-checking - others	
10.POTENTIAL LIMITATIONS	
Please list down Limitation	
Limitation reported/ Limitation discussed	
Limitation - others	
11.OTHER DETAILS	
Is this model an extension of another model (If yes, please mention what model it is)	
User interface	
Is this model a simulation software? (if yes, please mention the name of the software)	
Other comments	

Table S4. Occurrence of model outcome types (Some models included more than one output type).

Outcome type	Model name / First Author	Number of models
**Health economics outcome**	Baker model, Barnett model, BENESCO model, $BODE^3$, Cantor model, CDM, Chevreul model, CHD Policy model, ECEA tobacco tax model, EQUIPTMOD, Jiménez model, Johansson model, LCPM, Mendez model, ModelHeath: Tobacco, PRISM, Parrott model, Tobacco Town ABM, UKHF simulation	19
**Other health outcomes**	Barnett model, BENESCO model, $BODE^3$, Cantor model, CDM, CEPAC-US model, DYNAMO-HIA, ECEA tobacco tax model, EQUIPTMOD, IMPACT model, Johansson model, Mendez model, ModelHeath: Tobacco, Parrott model	14
**Mortality rate**	Barnett model, BENESCO model, CEPAC-US model, CHD Policy Model, DYNAMO-HIA, ECEA tobacco tax model, IMPACT model, LCPM, ModelHeath: Tobacco, PHIM, PRISM, SimSmoke	12
**Smoking prevalence**	Baker model, $BODE^3$, CDM, IMPACT, Jiménez model, LCPM, Mejia model, Mendez model, ModelHeath: Tobacco, UKHF simulation	10
**Morbidity rate**	Baker model, BENESCO model, DYNAMO-HIA, Mejia model, ModelHeath: Tobacco, PRISM, SimSmoke, UKHF simulation	8
**Equity**	$BODE^3$, CDM, ECEA tobacco tax model, IMPACT model	4
**Hospital admission**	ModelHeath: Tobacco	1

BENESCO model: Benefits of Smoking Cessation on Outcomes model
$BODE^3$: Burden of Disease Epidemiology, Equity and Economics model
CDM: Chronic Disease Model
CEPAC-US model: Cost-Effectiveness of Preventing AIDS Complications-US model
CHD Policy model: Coronary Heart Disease Policy model
LCPM: Lung Cancer Policy Model
PHIM: Population Health Impact Model
PRISM: Prevention Impacts Simulation Model
UKHF simulation: UK Health Forum simulation

Table S5.Occurrence of number of disease groups simulated by models.

Disease group	Model name / First Author	Number of models
**No disease explicitly modelled**	Baker model, Barnett model, Cantor model, Jiménez model, Mejia model, Mendez model, SimSmoke, Tobacco Town ABM	8
**One disease group**	CEPAC-US model, IMPACT, LCPM, PRISM	4
**Two disease groups**	CHD Policy model	1
**Three disease groups**	BODE^3, Chevreul model, DYNAMO-HIA, ModelHeath: Tobacco, PHIM	5
**Four disease groups**	BENESCO model, ECEA tobacco tax model, EQUIPTMOD, Johansson model, Parrott model, UKHF simulation, CDM	7

BENESCO model: Benefits of Smoking Cessation on Outcomes model
BODE^3: Burden of Disease Epidemiology, Equity and Economics model
CDM: Chronic Disease Model
CEPAC-US model: Cost-Effectiveness of Preventing AIDS Complications-US model
CHD Policy model: Coronary Heart Disease Policy model
LCPM: Lung Cancer Policy Model
PHIM: Population Health Impact Model
PRISM: Prevention Impacts Simulation Model
UKHF simulation: UK Health Forum simulation

Table S6. Diseases groups included in models.

Model name / First Author	Cancers	Chronic obstructive pulmonary disease	Cardiovascular disease	Other smoking-related diseases	No reported disease modelled
**SimSmoke**					Y - calculated smoking-attributable deaths
**BODE[3]**	Y		Y	Y	
**IMPACT**			Coronary heart disease (CHD)		
**ECEA tobacco tax model**	Y	Y	Y	Y	
**EQUIPTMOD**	Y	Y	CHD	Y	
**DYNAMO-HIA model**	Y	Y		Y	
**BENESCO model**	Y	Y	CHD	Y	
**Jiménez model**					Y
**Johansson model**	Y	Y	CHD and stroke	Y	
**PRISM**			Y		
**Baker model**					Y
**Barnett model**					Y - smoking related mortality risk
**Cantor model**					Y
**Chevreul model**	Y	Y	Y		
**CEPAC-US model**	Y				
**ModelHeath: Tobacco**	Y		Y	Y	
**Parrott model**	Y	Y	CHD	Y	
**PHIM**	Y	Y		Y	
**Tobacco Town ABM**					Y
**UKHF simulation**	Y	Y	CHD	Y	
**CDM**	Y	Y	CHD	Y	
**CHD Policy model**			CHD and stroke		
**LCPM**	Y				
**Mendez model**					Y
**Mejia model**					Y
**Total number**	**14**	**10**	**13**	**11**	**8**

BENESCO model: Benefits of Smoking Cessation on Outcomes model
BODE3: Burden of Disease Epidemiology, Equity and Economics model
CDM: Chronic Disease Model
CEPAC-US model: Cost-Effectiveness of Preventing AIDS Complications-US model
CHD Policy model: Coronary Heart Disease Policy model
LCPM: Lung Cancer Policy Model
PHIM: Population Health Impact Model
PRISM: Prevention Impacts Simulation Model
UKHF simulation: UK Health Forum simulation

Supplementary Table S7. Occurrence of model validation types (Some models used more than one validation type).

Validation type	Model name / First Author	Number of models
**No validation**	Baker model, Barnett model, BENESCO model, Cantor model, CDM, CHD Policy model, DYNAMO-HIA model, EQUIPTMOD, Mejia model, Parrott model, Tobacco Town ABM, UKHF simulation	12
**External validation**	BODE[3], CEPAC-US model, Chevreul model, IMPACT, LCPM, Mendez model, ModelHeath: Tobacco, PRISM, SimSmoke, Johansson model	10
**Internal validation**	CEPAC-US model, Chevreul model, Jiménez model, ModelHeath: Tobacco	4
**Cross validation**	BODE[3], CEPAC-US model	2

BENESCO model: Benefits of Smoking Cessation on Outcomes model
BODE3: Burden of Disease Epidemiology, Equity and Economics model
CDM: Chronic Disease Model
CEPAC-US model: Cost-Effectiveness of Preventing AIDS Complications-US model
CHD Policy model: Coronary Heart Disease Policy model
LCPM: Lung Cancer Policy Model
PHIM: Population Health Impact Model
PRISM: Prevention Impacts Simulation Model
UKHF simulation: UK Health Forum simulation

Table S8. Model score (in descending order of the number of peer-reviewed articles).

Model name / First Author	Population	Policy effectiveness	Smoking status	Smoking-related diseases	Lag time	Transparency	Sensitivity	Validation	Equity	Score	Number of publications*	Overall number of publications**
**SimSmoke**	1	0	1	0	1	1	1	1	0	**6**	18	**44**
**BODE[3]**	1	1	0	1	1	1	1	1	1	**8**	11	**11**
**IMPACT**	1	1	0	1	0	1	1	1	1	**7**	6	**6**
**ECEA tobacco tax model**	1	0	1	1	0	1	1	1	1	**7**	5	**5**
**EQUIPTMOD**	1	1	0	1	0	1	1	0	0	**5**	5	**5**
**DYNAMO-HIA model**	1	1	0	1	1	1	1	0	0	**6**	5	**5**
**BENESCO**	1	1	0	1	0	0	1	0	0	**4**	4	**20**
**Jiménez model**	1	1	1	0	0	0	1	1	0	**5**	3	**3**
**Johansson model**	0	1	0	1	1	1	1	1	0	**6**	3	**3**
**PRISM**	1	0	0	1	1	1	1	1	0	**6**	3	**3**
**Baker model**	1	1	0	0	1	0	1	0	0	**4**	2	**2**
**Barnett model**	0	1	0	0	0	1	1	0	0	**3**	2	**2**
**Cantor model**	0	1	0	0	0	0	1	0	0	**2**	2	**2**
**Chevreul model**	1	1	0	1	1	0	1	1	0	**6**	2	**2**
**CEPAC-US model**	1	1	1	1	1	1	1	1	0	**8**	2	**2**
**ModelHeath: Tobacco**	1	1	0	1	1	1	1	1	0	**7**	2	**2**
**Parrott model**	0	1	0	1	0	1	1	0	0	**4**	2	**2**
**PHIM**	1	0	0	1	1	1	1	1	0	**6**	2	**2**
**Tobacco Town ABM**	1	1	1	0	0	1	1	0	0	**5**	2	**2**
**UKHF simulation**	1	1	0	1	1	1	1	0	0	**6**	2	**2**
**CDM**	1	1	0	1	0	1	1	0	1	**6**	1	**7**
**CHD Policy model**	1	1	0	1	0	0	1	0	0	**4**	1	**2**
**LCPM**	1	1	1	1	0	1	1	1	0	**7**	1	**2**
**Mendez model**	1	0	0	0	0	1	1	1	0	**4**	1	**5**
**Mejia model**	1	1	0	0	0	1	1	0	0	**4**	1	**2**
**Number of models (%)**	**21 (84%)**	**20 (80%)**	**6 (24%)**	**17 (68%)**	**11(44%)**	**19 (76%)**	**25 (100%)**	**13 (52%)**	**4 (16%)**			

* Search period between July 2013 to August 2019

** Search period before August 2019
BENESCO model: Benefits of Smoking Cessation on Outcomes model
BODE3: Burden of Disease Epidemiology, Equity and Economics model
CDM: Chronic Disease Model
CEPAC-US model: Cost-Effectiveness of Preventing AIDS Complications-US model
CHD Policy model: Coronary Heart Disease Policy model
LCPM: Lung Cancer Policy Model
PHIM: Population Health Impact Model
PRISM: Prevention Impacts Simulation Model
UKHF simulation: UK Health Forum simulation

Figure S1. Model score and number of peer-reviewed publications* linked to the model.

The slope remained positive even after removing SimSmoke and BENESCO models

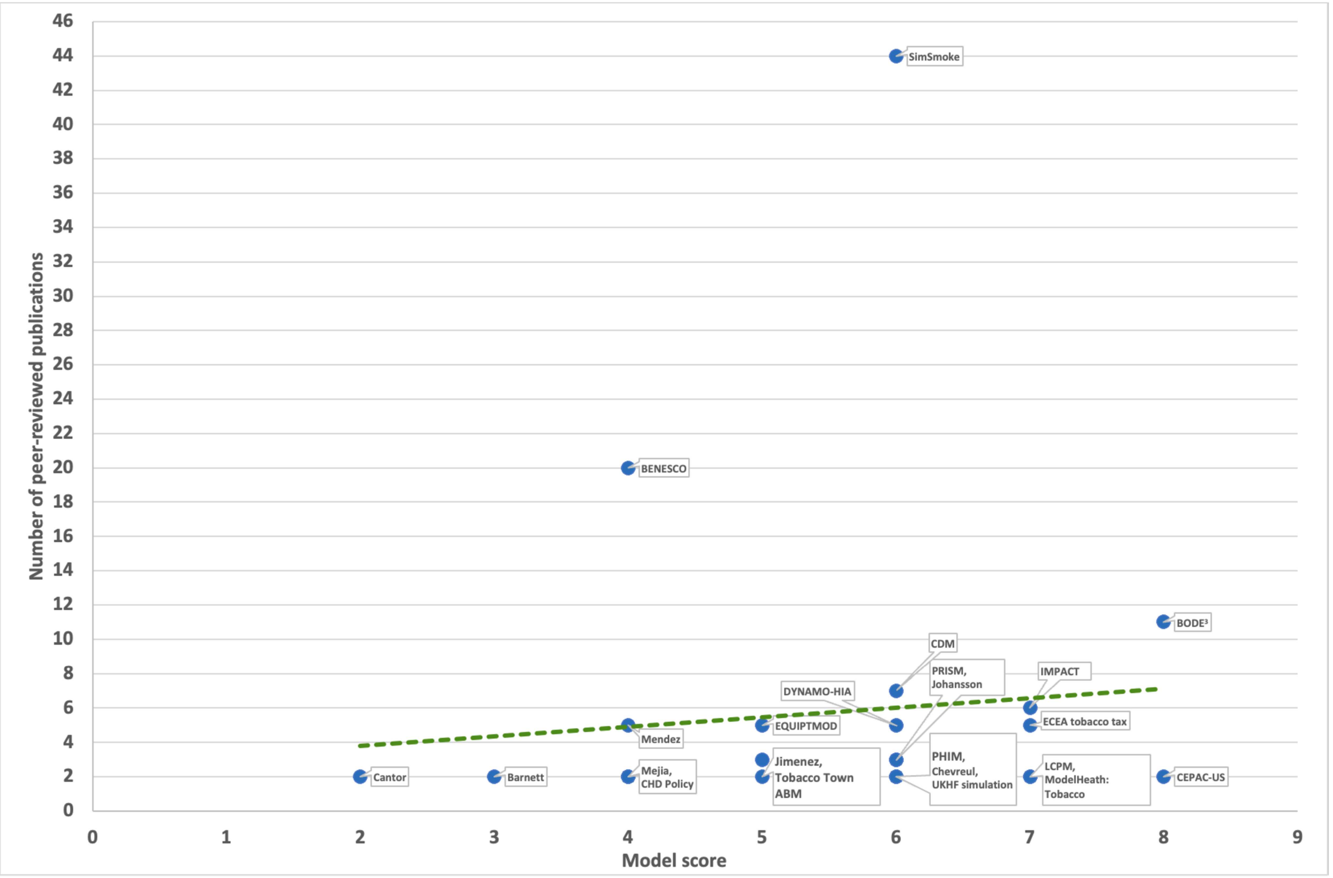

* Search period before August 2019

BENESCO model: Benefits of Smoking Cessation on Outcomes model
BODE3: Burden of Disease Epidemiology, Equity and Economics model
CDM: Chronic Disease Model
CEPAC-US model: Cost-Effectiveness of Preventing AIDS Complications-US model
CHD Policy model: Coronary Heart Disease Policy model
LCPM: Lung Cancer Policy Model
PHIM: Population Health Impact Model
PRISM: Prevention Impacts Simulation Model
UKHF simulation: UK Health Forum simulation
